# VMamba for plant leaf disease identification: design and experiment

**DOI:** 10.3389/fpls.2025.1515021

**Published:** 2025-04-02

**Authors:** Hewei Zhang, Shengzhou Li, Jialong Xie, Zihan Chen, Jiyang Chen, Jianwen Guo

**Affiliations:** School of Mechanical Engineering, Dongguan University of Technology, Dongguan, China

**Keywords:** plant leaf disease, VMamba, transformer, diffusion model, transfer learning, few-shot learning

## Abstract

**Introduction:**

The rapid spread of crop diseases poses a severe threat to agricultural production, significantly reducing both the yield and quality of crops. In recent years, plant disease recognition technologies based on machine vision and artificial intelligence have made significant progress. However, current mainstream deep learning architectures still face numerous challenges in detecting agricultural plant diseases. These include issues such as the complexity of agricultural environments and the reduced accuracy and increased training time caused by small sample sizes of agricultural plant diseases.

**Methods:**

To address these challenges, we introduce the VMamba visual backbone model into the task of detecting agricultural plant diseases. This model effectively reduces computational complexity through a selective scanning mechanism while significantly improving classification accuracy by maintaining a global receptive field and leveraging dynamic weighting advantages. Our study proposes the DDHTLVMamba method, which combines VMamba with diffusion models and transfer learning techniques, and applies it to the detection of plant diseases in small-sample agricultural datasets. This research evaluates the performance of VMamba across different datasets and training methods, conducting comparative analyses with mainstream deep learning architectures.

**Results and discussion:**

Experimental results demonstrate that the VMamba model outperforms mainstream models such as ResNet50, Vision Transformer, and Swin Transformer in disease recognition accuracy, whether on large-scale datasets like PlantVillage or optimized small-sample disease datasets, showcasing superior performance. Compared to Swin Transformer, VMamba achieves a 3.49% increase in accuracy while reducing training time by 80%. Furthermore, the proposed DDHTLVMamba training method demonstrates its effectiveness on small-sample datasets, significantly reducing pre-training time while maintaining recognition accuracy comparable to that achieved with large-sample transfer learning. This study provides an innovative approach for the efficient identification of agricultural diseases and is expected to advance the development of intelligent agricultural disease prevention and control technologies.

## 1 Introduction

Crop diseases pose a serious threat to agricultural production, typically caused by bacteria, fungi, microorganisms, or viruses (Sankaran et al., 2010). Once a disease occurs, it spreads rapidly, often leading to significant decreases in crop yield and quality (Thomas et al., 2018). Therefore, accurate and rapid identification and classification of crop diseases are crucial for effective disease prevention (Shoaib et al., 2023). The physiological state of plants can often be reflected through the leaves (Joshi and Bhavsar, 2020), and changes in leaves accurately indicate the growth conditions of plants and potential diseases. In recent years, significant progress has been made in leaf disease identification using machine vision and artificial intelligence. These technologies reduce the need for operators to have specialized knowledge in agricultural disease analysis, thereby reducing learning costs and labor consumption, while being easy to transfer and widely applicable (Li et al., 2021). In particular, deep learning visual models have excelled in handling complex inputs and classification tasks, having been successfully applied to disease identification in crops such as maize, wheat, citrus, and potatoes (Lee et al., 2020).

However, images of agricultural plant diseases often present challenges like complex backgrounds and unclear diseased areas, especially when sample sizes are insufficient, resulting in a significant drop in recognition performance, which poses a major challenge for computer vision (Mohanty et al., 2016). The introduction of deep residual networks (ResNet) addressed the gradient vanishing problem in deep networks, making it possible to construct deeper learning architectures (He et al., 2016). However, training and inference with ResNet require substantial computational resources, and there is still considerable redundancy within the network. In contrast, the Vision Transformer (ViT) adopts a self-attention mechanism that can process all positions in the input sequence in parallel, significantly improving training speed and inference efficiency (Dosovitskiy et al., 2020). However, with an increase in input scale, the computational complexity of ViT grows quadratically, especially when dealing with high-resolution tasks, leading to significant computational costs, while in agricultural applications, a balance between performance and efficiency is essential.

The VMamba visual backbone model, based on state space models (SSMs) and a selective scanning mechanism, provides an alternative solution to ViT for computer vision (Gu and Dao, 2023). The selective scanning mechanism in the VMamba model effectively reduces the complexity of attention computation. Compared to the ViT model, VMamba maintains the advantages of global effective receptive fields (ERF) and dynamic weights while achieving linear computational complexity and demonstrating excellent classification accuracy (Liu et al., 2024). Therefore, we aim to leverage the strengths of VMamba in the complex task of agricultural plant disease identification to not only maintain high recognition accuracy but also effectively reduce computational resource and data requirements.

The main objective of this paper is to introduce the VMamba visual backbone model into the field of agricultural plant disease identification, investigating its effectiveness in crop disease identification. Specifically, this study focuses on: (1) verifying the performance advantages of the VMamba model in agricultural disease classification; (2) exploring the effectiveness of VMamba in small-sample plant disease identification and its performance in complex crop growth environments; (3) examining the transfer learning performance of VMamba in agricultural disease identification, in combination with diffusion models and transfer learning; and (4) designing the DDHTL-VMamba model to further enhance recognition performance in complex agricultural environments through diffusion models and transfer learning, providing a new approach for agricultural disease prevention.

The remainder of this paper is organized as follows: Section 2 introduces related research work; Section 3 presents the proposed method; Section 4 introduces the experiments; Section 5 presents related discussions; and Section 6 provides the conclusions and future work.

## 2 Related works

The identification and classification of plant leaf images are considered effective means for disease identification (Shoaib et al., 2023). Hossain et al. divided the machine learning identification process of plant leaves into three steps: image preprocessing, feature extraction, and classification (Hossain et al., 2019). However, plant disease images often feature complex backgrounds and indistinct disease areas, increasing the difficulty of feature extraction and classification. Due to labor-intensive feature extraction, slow data accumulation, and weak generalization ability, traditional machine learning-based plant disease identification methods face significant challenges in dealing with complex disease data (Lee et al., 2020).

With the development of deep learning, Geetharamani et al. proposed a technique with higher accuracy than traditional machine learning methods, providing a new solution for crop disease identification (Geetharamani and Pandian, 2019). Zhang et al. proposed a cucumber leaf disease identification technique based on convolutional neural networks (CNN) (Zhang et al., 2019), and Tang et al. achieved good results in grape disease image classification using CNN (Tang et al., 2020). Kamal et al. widely applied popular deep learning image classification techniques like CNN to crop disease identification, significantly improving the efficiency of disease identification (Kamal et al., 2019). Even in cases with complex backgrounds and indistinct disease targets, deep learning-based models still demonstrate excellent generalization capabilities. However, CNN primarily relies on local receptive fields, making it difficult to model global information, which leads to certain limitations in feature extraction.

ViT is a deep learning-based image classification technology capable of learning high-quality intermediate features while preserving more spatial information compared to ResNet (Dosovitskiy et al., 2020). In the field of leaf disease image recognition, ViT more effectively selects areas with significant features through patch segmentation, allowing its attention mechanism to better identify disease regions and accurately determine disease types (Fu et al., 2024). Thakur et al. applied ViT to plant leaf disease identification and demonstrated that it can effectively identify various diseases in multiple crops and, due to its lightweight structure, shows significant performance advantages in plant disease classification tasks (Thakur et al., 2023). Borhani et al. combined CNN and ViT for plant disease identification, further improving prediction speed while maintaining high accuracy (Borhani et al., 2022). However, the self-attention mechanism of ViT results in computational complexity that is quadratically related to the length of the input sequence. Specifically, for an image divided into N patches, the computational complexity of the self-attention mechanism is O(N²), meaning that when the image resolution increases, the computational load rises sharply. Saleem et al. proposed a new hybrid architecture with enhanced vegetative feature isolation, combining deep learning models to improve the performance of multi-crop disease detection, providing important reference for this study (Saleem et al., 2024). Naeem et al. used feature selection and feature concatenation methods to optimize potato leaf disease identification tasks, while our method employs state space models (SSM) for feature extraction to further reduce computational complexity (Naeem et al., 2025). ViT models require an ultra-large-scale dataset for training. When training data is insufficient, its low-level attention mechanism cannot learn local information, leading to reduced model accuracy, limiting ViT’s performance on small-sample datasets (Zhao, 2017).

Swin Transformer, through a local window attention mechanism (Liu et al., 2021), associates each position with only pixels within its local window, thereby reducing the complexity of attention computation. Swin Transformer can learn and detect features at different scales, adapting to targets of different scales, thus demonstrating strong performance in tasks such as image classification, object detection, and semantic segmentation. Zhang et al. applied Swin Transformer to field rice disease identification and significantly improved the accuracy of disease detection compared to other popular models (Zhang et al., 2021). However, due to its local window computation approach, Swin Transformer may lead to the loss of information from certain diseased areas, limiting its performance in agricultural disease recognition tasks with complex environmental backgrounds. Additionally, when processing high-resolution images, its self-attention mechanism still causes a sharp increase in computational load.

In recent years, few-shot learning (FSL) has gained significant attention in agricultural disease detection tasks. PDSE-Lite extracts global features through a convolutional autoencoder (Bedi et al., 2024), improving the accuracy of disease severity estimation. However, its ability to model fine-grained lesions (e.g., vein yellowing caused by Huanglongbing) is limited. Saleem et al. proposed a multi-scale feature extraction and fusion method, optimizing few-shot learning models for wheat disease classification problems, providing inspiration for our approach (Saleem et al., 2025). TrIncNet introduced a lightweight ViT architecture optimization scheme to adapt to low-data environments, but its attention mechanism still requires O(N²) computational complexity, making it difficult to handle high-resolution field images (Gole et al., 2023). Furthermore, Hybrid CNN-Autoencoder combines CNNs and autoencoders, enhancing classification accuracy in scenarios with limited data (Bedi and Gole, 2021). Existing methods predominantly rely on large-scale pre-trained datasets such as ImageNet, while the few-shot generalization capability in agricultural disease scenarios remains insufficiently validated.

VMamba is a visual backbone network based on state space models (SSMs). Unlike traditional attention mechanisms, SSMs allow each element in the sequence to interact with previously scanned samples, reducing the computational complexity of attention from quadratic to linear (Shi et al., 2024). The core advantage of VMamba is incorporating SSMs’ global receptive fields, input-dependent weighting parameters, and linear computational complexity into visual processing. In image classification tasks, VMamba outperforms popular CNN and ViT models while maintaining linear computational complexity (Liu et al., 2024). Chen et al. applied VMamba to fine-grained food visual classification tasks, combining it with residual networks, and demonstrated its superiority on high-resolution datasets, outperforming existing state-of-the-art (SOTA) models on ImageNet (Chen C. S. et al., 2024). Dang et al. used VMamba for medical image segmentation tasks, achieving significantly better performance in multiple tasks than CNN- and Transformer-based models (Dang et al., 2024).

## 3 Methods

### 3.1 State space models

State space models (SSMs) are mathematical frameworks for characterizing dynamic systems, establishing a mapping relationship between input signals and output signals by modeling the evolution of hidden states. Their core concept originates from linear time-invariant systems (LTI) and can be described using linear ordinary differential equations (ODEs), as shown in Equation 1.


(1) $$\left\{ \begin{array}{c} h'(t)=Ah(t)+Bu(t)\\ y(t)\;=Ch(t)+Du(t) \end{array}\right.$$

The terminology definitions are as follows:

$h(t)\in R^{N}$ : The hidden state at time t, which carries the historical information of the system.

u(t)∈R : The input signal that drives the evolution of the state.

y(t)∈R : The output signal, which is jointly determined by the current state and the input.

$A\in R^{N\times N}$ : The state transition matrix, which governs the evolution of the hidden state.

$B\in R^{N\times 1}$ : The input projection matrix, which maps the input to the state space.

$C\in R^{1\times N}$ : The output projection matrix, which maps the state to the output.

$D\in R^{1}$ : The direct transmission term (usually set to 0 to simplify the model) (Liu et al., 2024).

In deep learning, the required state transitions are discrete rather than continuous, so the above linear ordinary differential equations must be converted into discrete functions. Considering an input as a d-dimensional signal stream of length L, this paper employs the Zero-Order Hold (ZOH) method to discretize the aforementioned differential equation. It assumes that the input remains constant over the time interval Δ and solves the ODE through integration, yielding the following discrete recurrence relation, as shown in Equation 2:


(2) $$h_{k}=\bar{A}h_{k-1}+\bar{B}u_{k}$$

where the discretization parameters are defined as:


$$\bar{A}=e^{A\text{Δ}},\;\bar{B}=(\int_{0}^{\text{Δ}}e^{A(\text{Δ}-\tau )}d\tau )B\approx \Delta B$$

The Zero-Order Hold (ZOH) method is a standard technique used to approximate continuous-time systems in discrete form. Its purpose is to ensure that the behavior of the discretized system closely resembles that of the original continuous-time system. Specifically, ZOH assumes that the input signal remains constant within the sampling interval, thereby simplifying the computation of hidden state updates. The advantage of this approach lies in its ability to accurately capture the dynamics of the input signal while maintaining linear computational complexity, ensuring numerical stability for high-resolution image inputs.

### 3.2 Selective scanning mechanism

The static parameters A , B , and C in traditional State Space Models (SSMs) limit their adaptability to context. To address this, the Selective Scanning Mechanism (S6) introduces input-dependent dynamic parameters: $B_{k}\;,\;C_{k}\;,{\text{ Δ}}_{k}\;=f_{\theta }(u_{k})$ . The selective scanning mechanism (called S6) allows the model to selectively process information to focus on or ignore specific inputs. S6 transforms the dimensions of the matrices affecting the input B and the state-influencing C to (B, L, N), where these parameters correspond to batch size, sequence length, and hidden state size, respectively, and changes the size of Δ to (B, L, D). This means that during inference, the model can dynamically calculate the values of matrices $B_{k}$ , $C_{k}$ , and step length ${\text{ Δ}}_{k}$ based on different input data, thus achieving the goal of selectively retaining content in the hidden state and ignoring certain content (Gu and Dao, 2023). In S6, for the input sequence $u_{1}$ , $u_{1}$ ,…, $u_{T}$ , the hidden state is updated as follows, as shown in Equation 3:


(3) $$h_{k}=w_{k}⊙h_{k-1}+\sum_{i=1}^{k}(\prod_{j=i}^{k}e^{-A_{j}{\text{Δ}}_{j}})B_{i}u_{i}$$

Here, $w_{k}=e^{A_{k}{\text{Δ}}_{k}}$ represents the dynamic decay weight, and ⊙ denotes element-wise multiplication.

For an input feature map $X\in R^{H\times W\times C}$ , the output is given by Equation 4:


(4) $$Y=\sum_{d\in \{Four\;directions\}}\text{Reshape}(\text{S}6_{d}({\text{Flatten}}_{d}(X)))$$

**Figure 1:**
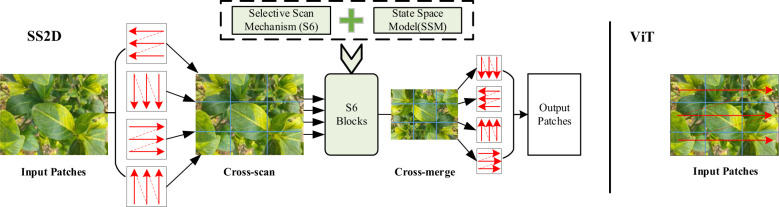
SS2D architecture.

In the VMamba architecture, the Selective Scanning Mechanism is applied within the SS2D module. To account for the two-dimensional nature of visual data, a Cross Scan Module (CSM) is used to generate sequential inputs. The input sequence is expanded along multiple scanning paths, extending them into sequences along rows and columns (cross-scanning). Subsequently, the sequences are scanned in four different directions. Cross-Scan: This process divides the input image data into multiple patches and unfolds them along four distinct scanning paths (horizontal, vertical, and two diagonal directions). This allows each patch to interact with others in different orders, thereby collecting contextual information from various directions. Parallel Processing: Parallel processing employs S6 Blocks, which are modules based on State Space Models (SSMs). In each scanning path, the S6 module is responsible for selectively weighting and processing each image patch. The S6 module dynamically adjusts the weight of each patch through an input-dependent selection mechanism, enabling the model to focus on important regions. Cross-Merge: After the four scanning paths complete their respective processing, the Cross-Merge block combines the results from the four scanning paths into a final two-dimensional feature map. This process integrates information from the four different scanning directions, ensuring that each patch receives contextual information from all directions. This is illustrated in **Figure 1**.

Transformer-based models like ViT divide the input image into smaller patches, flatten these patches, and feed them as sequences into the Transformer model (Dosovitskiy et al., 2020), as shown in **Figure 1**. This approach inevitably leads to a limited receptive field. Moreover, in the self-attention mechanism, each input element interacts computationally with all other elements, resulting in a quadratic computational complexity of O(N²), where N is the length of the input sequence. Specifically, ViT’s self-attention mechanism computes the similarity between every pair of input elements, leading to high computational and memory costs when handling high-resolution images.VMamba adopts the Selective Scan mechanism, allowing each input element to interact only with its neighboring elements, thereby reducing the computational complexity to linear. This approach not only enhances computational efficiency but also maintains a global receptive field, ensuring the model’s effectiveness in handling visual tasks.

### 3.3 VMamba architecture

**Figure 2:**
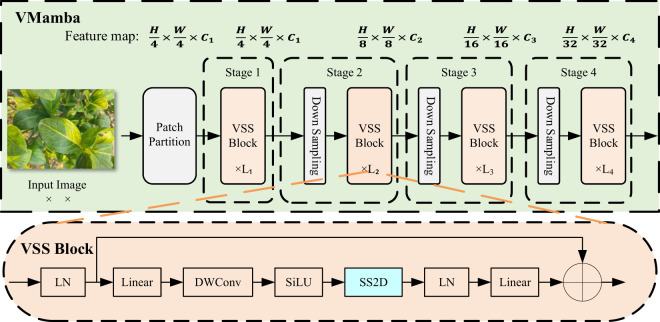
VMamba architecture.

The VMamba architecture is shown in **Figure 2**, where all these stages together build a hierarchical representation similar to CNN and ViT models (Liu et al., 2024). The stages are described as follows: Patch Partition: Input images are divided into multiple patches using a Patch Partition similar to ViT (Dosovitskiy et al., 2020), obtaining a feature map of size. **Stage 1**: Several VSS blocks are stacked on the feature map, maintaining the same dimensions. **Stage 2**: The feature map from Stage 1 is downsampled, and more VSS Blocks are stacked to produce an output resolution of. **Stage 3 & Stage 4**: Repeat the process of Stage 2 to create feature maps with resolutions of and, respectively.

The VSS Block is the core of the VMamba architecture. In the VSS Block, the input is passed through Layer Normalization (LN), and the output is split into two streams of information. One stream passes through a 3x3 Depthwise Convolution (DWConv) layer (Chollet, 2017), followed by SiLU activation and entering the core SS2D module. The SS2D output is then passed through a normalization layer and added to the output of the other stream.

SS2D is the key element of the VSS Block, integrating the S6 Block with the Cross Scan Module (CSM), as shown in **Figure 1**. To address the limitations of LTI SSMs in capturing contextual information, the S6 Block combines S6 with SSMs, allowing each element in a 1D array to interact with any previously scanned sample by compressing the hidden state, effectively reducing quadratic complexity to linear (Liu et al., 2024). VMamba incorporates the selective scanning mechanism (S6) as the core SSMs operator, processing input data causally, thereby capturing information only within the scanned portion of the data. Although the order of operations in the S6 Block aligns with NLP tasks involving temporal data, it faces significant challenges when applied to non-temporal data (e.g., images, graphs, sets) because visual sequences are inherently non-sequential (Liu et al., 2024). To solve this problem, Chen et al. proposed the Cross Scan Module (CSM). CSM selects image patches, expands them into sequences along rows and columns (cross-scan), and then scans in four different directions: top-left to bottom-right, bottom-right to top-left, top-right to bottom-left, and bottom-left to top-right (Chen C. S. et al., 2024). This allows any pixel to integrate information from all other pixels in different directions. Each sequence is independently processed by different S6 blocks, then reshaped into a single image, and all sequences are merged into a new sequence (cross-merge).

In the SS2D Block, input blocks are traversed along four different scanning paths (cross-scan), each sequence is independently processed by different S6 blocks, and the results are subsequently fused to construct a 2D feature map (cross-merge) as the final output. Data forwarding in the SS2D Block is divided into three steps: cross-scan, S6 block selective scanning, and cross-merge. The SS2D Block allows visual data to use 1D selective scanning, processes image blocks in parallel through S6 blocks, and merges the results to form a 2D feature map, thereby effectively utilizing the S6 module. It inherits the linear complexity of the selective scanning mechanism while retaining a global receptive field (Liu et al., 2024).

### 3.4 Denoising diffusion probabilistic models

In deep learning image classification tasks, data augmentation techniques are widely used to address the issue of insufficient training data. By applying transformations such as rotation, translation, scaling, cropping, flipping, and adding Gaussian or salt-and-pepper noise to the original data, the diversity of the data is increased, thereby enhancing the model’s generalization ability and robustness. However, traditional data augmentation methods have certain limitations. These methods typically rely on manually designed rules, which may fail to fully capture the complex features of the data, resulting in limited diversity of the generated samples and an inability to cover the full scope of the data distribution. Moreover, excessive reliance on data augmentation can lead to overfitting of the model to specific transformations, affecting its performance on unseen samples. To overcome these limitations, DDPM offers a new approach to image augmentation (Ho et al., 2020).

**Figure 3:**
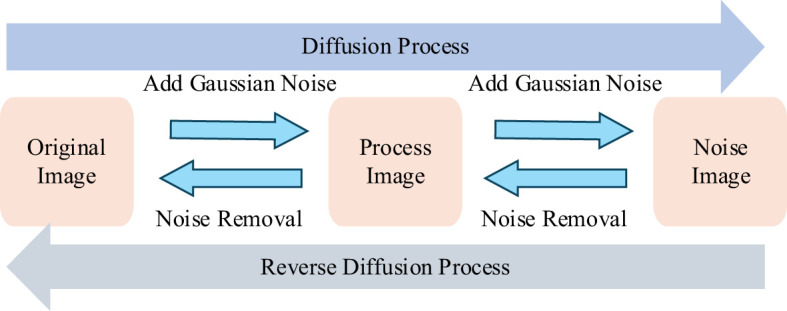
Diffusion process.

**Figure 4:**
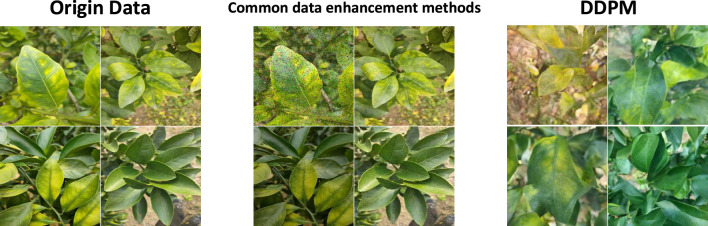
Comparison of data enhancement methods.

Denoising Diffusion Probabilistic Models (DDPM) are latent variable models inspired by non-equilibrium thermodynamics. The structure of DDPM is shown in **Figure 3**. DDPM defines a Markov chain for the diffusion steps, gradually adding random noise to the data, and then learns the reverse diffusion process to reconstruct the desired data sample from noise, thus generating target data samples from noise (Ho et al., 2020). The diffusion model consists of two main processes: the forward process (diffusion process) and the reverse process. By training a DDPM diffusion model to learn the diffusion of image data, we input randomly generated noisy images into the DDPM diffusion model and perform the reverse diffusion process to ultimately obtain synthesized images similar to real images. A visual comparison between images generated by DDPM and traditional image generation methods is shown in **Figure 4**.

Unlike other generative models, such as Variational Autoencoders (VAEs) and Generative Adversarial Networks (GANs), diffusion models gradually add noise to images during the forward process until the image is completely destroyed, becoming Gaussian noise. During the reverse process, the model learns how to recover the original image from Gaussian noise (Li et al., 2023). This stepwise noise addition and denoising process make diffusion models exhibit superior stability and diversity when generating samples, making them an important focus of recent generative model research.

### 3.5 Transfer learning

Transfer learning (Zhuang et al., 2020) is a machine learning technique that applies knowledge learned from one domain (source domain) to another related domain (target domain) to improve model performance on a new task. This method is especially suitable for situations with limited data, as it leverages existing knowledge to reduce the amount of training data required for new tasks. Transfer learning offers the advantage of saving time and computational resources, as it avoids the need to train a model from scratch, particularly for complex tasks. Moreover, pretrained models provide better initial weights, accelerating convergence and improving accuracy (Yao et al., 2020). The main steps of transfer learning are as follows: (1) Pretraining: A deep learning model is trained on a large dataset (e.g., ImageNet) to learn general features. For instance, convolutional neural networks (CNNs) can extract edges, textures, and other low-level features from images. (2) Feature Transfer: The weights and structure of the pretrained model are transferred to the target task. The model is fine-tuned on a small amount of data to adjust its parameters. (3) Evaluation and Optimization: The transferred model is evaluated using data from the target domain and further optimized as needed.

### 3.6 Integration of DDPM, transfer learning, and VMamba for disease identification

To evaluate the performance and characteristics of the VMamba model in recognizing plant leaf diseases, we designed five methods that integrate DDPM, transfer learning, and VMamba.

**Method 1:** A Plant Disease Recognition Method Using VMamba (Large Image Dataset). This method applies the VMamba model to a large dataset of plant leaf diseases to evaluate its performance on a big dataset.

**Method 2:** A Plant Disease Recognition Method Using VMamba (Small Image Dataset). This method uses the VMamba model on a small sample dataset of plant leaf diseases, testing its performance with limited data.

**Figure 5:**
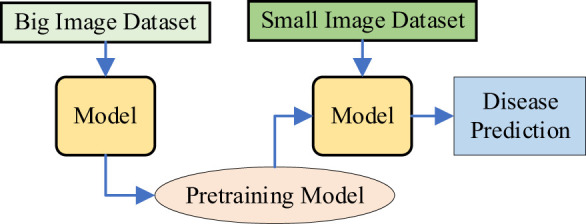
Transfer learning-based plant disease recognition method using VMamba.

**Method 3:** A Transfer Learning-Based Disease Recognition Method with VMamba. As shown in **Figure 5**, this method first pre-trains the VMamba model on a large-scale plant disease dataset, generating a pre-trained model. Transfer learning is then used to transfer the learned network architecture and weights to the small sample dataset for recognizing diseases in plant leaf images with limited data.

**Figure 6:**
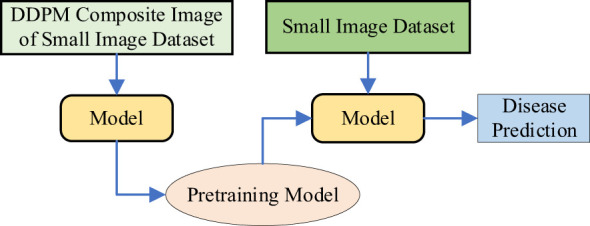
The workflow of method 4.

**Method 4:** Diffusion-Driven Transfer Learning with VMamba (Small Image Dataset). As shown in **Figure 6**, this method combines DDPM and transfer learning for VMamba on small datasets. DDPM is used to augment the small plant disease dataset by generating additional images, thereby increasing the training data and enhancing the model’s generalization. The model trained on the augmented images is used as a pre-trained model. Transfer learning (Zhuang et al., 2020) is then applied to fine-tune this pre-trained model on the original small sample dataset, aiming to improve the robustness of the VMamba model, reduce sensitivity to image variations, and ultimately improve accuracy.

**Figure 7:**
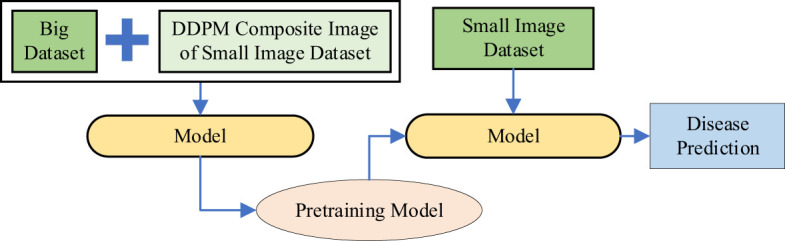
The Workflow of Method 5.

**Method 5:** Diffusion-Driven Hybrid Transfer Learning with VMamba (DDHTL-VMamba). As shown in **Figure 7**, this method consists of two stages:

1. **Pre-training Stage**: In this stage, DDPM (Denoising Diffusion Probabilistic Models) is used to generate an augmented image dataset based on a small-sample dataset (In-field small dataset). This augmented dataset is then combined with a larger dataset (PlantVillage dataset) for pre-training the model. The goal of this stage is to leverage the generative capabilities of DDPM to enrich the diversity of the training data, thereby improving the model’s ability to learn robust features.
2. **Transfer Learning Stage**: After pre-training, the model is fine-tuned on a small-sample plant disease dataset using transfer learning techniques. This involves adapting the pre-trained model to the specific characteristics of the target dataset, which typically contains limited labeled data. By doing so, the model can better capture the nuances of the target task while retaining the generalization benefits gained during pre-training.

The purpose of this approach is to enhance the accuracy and performance of the VMamba model in identifying plant diseases from small-sample datasets, as well as to improve its generalization capability in real-world application scenarios. This hybrid strategy effectively addresses the challenges posed by limited data availability, ensuring that the model performs reliably even when trained on smaller, domain-specific datasets.

## 4 Experiment

### 4.1 Experimental datasets and environment

In this study, we utilized the PlantVillage dataset as the large dataset and the In-field small dataset constructed by our team (Li et al., 2023; Chen et al., 2024a; Chen et al., 2024b) as the small sample dataset.

PlantVillage dataset consists of 54,303 images of healthy and diseased plant leaves, covering 38 different species and diseases. This includes crops such as tomatoes, apples, bananas, and their related diseases. This dataset provides a diverse set of images, creating an ideal environment for evaluating the performance of models in plant disease recognition tasks.

In-field small dataset is a small sample dataset focused on citrus plant diseases, collected from real-world field environments. It contains a total of 3,250 high-resolution (4000×3000) color images, divided into three categories: HLB-infected leaves (758 images), magnesium-deficient leaves (739 images), and healthy leaves (1,151 images). This dataset is particularly useful for testing the model’s performance in small-sample plant disease recognition tasks.

### 4.2 Performance evaluation metrics

**Figure 8:**
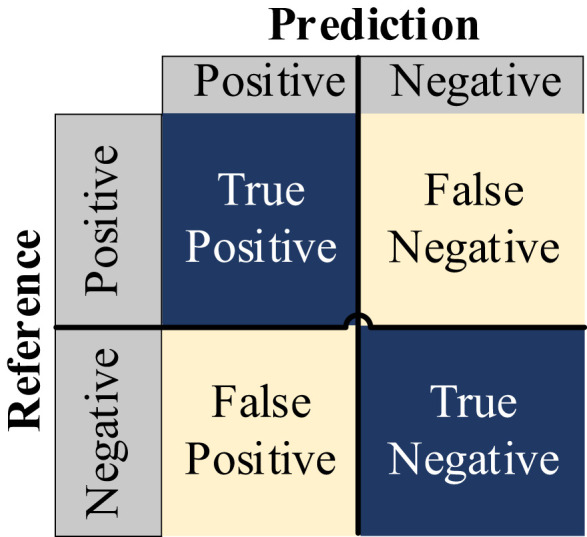
Confusion matrix.

The performance of the models was evaluated using several key metrics, including Params, Gflops, Time per epoch, AccTop1, AccTop5, Precision, Recall, and F1 Score, as detailed in **Table 1**. Additionally, Confusion Matrix analysis was employed in some experiments to further assess the classification performance of the models, ensuring accuracy in the results, as shown in **Figure 8**. The Confusion Matrix is a tool for evaluating classification model performance by visualizing the relationship between predicted and true labels. It helps analyze how well the model classifies different categories. Typically presented as a square matrix, each row represents the true class, and each column represents the predicted class, as shown in **Figure 8**. In this matrix, True Positive (TP) refers to the correctly predicted positive samples, True Negative (TN) refers to correctly predicted negative samples, False Positive (FP) represents the negative samples incorrectly predicted as positive (false alarms), and False Negative (FN) represents the positive samples incorrectly predicted as negative (missed detections).

**Table 1:** Evaluation metrics.

Evaluation Metrics	Description
Params	The number of parameters in the model, typically used to assess the model’s complexity and capacity. A higher number of parameters generally indicates greater model complexity, but it may also lead to overfitting.
Gflops	Gflops (Giga Floating Point Operations Per Second), measures the computational complexity and processing speed of the model, reflecting its computational efficiency.
Time per epoch	The time required for each training epoch, often used to evaluate the efficiency of the training process. Shorter times indicate that the model can iterate through training more quickly.
Acc-Top1	The accuracy of the model in correctly identifying the top predicted class in classification tasks. Higher values indicate better model performance.
Acc-Top5	The accuracy of the model in correctly identifying one of the top five predicted classes in classification tasks, reflecting the model’s robustness in multi-class recognition.
Precision	Precision measures the proportion of correctly predicted positive samples out of all samples predicted as positive. A high precision indicates the model’s strong accuracy in predicting the positive class.
Recall	Recall measures the proportion of actual positive samples that were correctly predicted as positive. A high recall indicates the model’s effectiveness in identifying positive samples.
F1 Score	The F1 Score is the harmonic mean of precision and recall, providing a balanced evaluation of the model’s performance, especially in cases of class imbalance. A higher F1 score indicates better overall performance.

### 4.3 Experiments

The experiments conducted in this study are summarized in **Table 2**. To ensure data consistency and facilitate model processing, all images were resized to a uniform resolution of 224×224 pixels during the data preprocessing stage of model training. Additionally, random cropping was applied to the input images within a specified range to augment the dataset and improve model robustness.The dataset was split into training, validation, and test sets in a 6:2:2 ratio, ensuring that the training and validation conditions remained consistent across different datasets. All computations were performed on the same hardware and operating environment to ensure controlled variables. The setup includes: CPU: R9 7940H, RAM: 16 GB, GPU: RTX 4060 Max-Q 8 GB, SYSTEM: Ubuntu 22.04, Python: 3.10, PyTorch: 2.0.0, and CUDA: 11.8. In terms of hyperparameter settings, we used a batch size of 16 for the VMamba network. A linear learning rate scaling strategy was employed for scheduling, and the AdamW optimizer was utilized for training. The number of training epochs was uniformly set to 300, and an Early-Stop mechanism was implemented to prevent overfitting and optimize training efficiency. This systematic approach ensures that the experimental results are reliable, reproducible, and reflective of the model’s true performance.

**Table 2:** Experiments.

Experiment	Objective	Method	Dataset
Experiment 1	Evaluating VMamba’s Disease Identification Ability	Method 1	Big Image Dataset PlantVillage
Experiment 2	Evaluating VMamba’s Disease Identification Ability on Small Image Dataset	Method 2	Small Image Dataset In-field small dataset
Experiment 3	Evaluating the Performance of VMamba Integrated with Transfer Learning	Method 3	same as above
Experiment 4	Evaluating the Performance of VMamba Integrated with DDPM and Transfer Learning	Method 4	Big Image Dataset PlantVillage; Small Image Dataset In-field small dataset
Experiment 5	Evaluating the Performance of DDHTL-VMamba	Method 5	same as above

#### 4.3.1 Results and analysis of experiment 1

The results of Experiment 1 are shown in **Table 3**. The findings demonstrate that the VMamba model outperformed other models across various metrics, including Acc-Top1, Acc-Top5, Precision, Recall, and F1 Score. Notably, VMamba achieved an Acc-Top1 accuracy of 99.81%, with the Swin Transformer coming in second at 99.18%. Furthermore, VMamba exhibited significant advantages in training speed, especially when compared to the ViT model, with much shorter training times per epoch.

**Table 3:** Experiment 1 results.

Model	VMamba	ResNet50	DeiT	ViT	Swin Transformer
Params	30.7M	23.59M	5.71M	85.68M	27.53M
Gflops	4.85	4.13	1.08	16.86	4.37
Acc-Top1 (%)	99.81	97.5	99.10	97.28	99.18
Acc-Top5 (%)	99.99	99.91	99.97	99.91	99.98
Precision (%)	99.51	96.06	98.65	96.61	98.88
Recall (%)	99.20	96.50	98.58	96.71	98.76
F1 Score (%)	99.34	96.21	98.60	97.29	98.81
Time per epoch	287s	237s	57s	655s	336s

The results show that although the ViT model has the highest parameter count (85.68M) and computational complexity (16.86 Gflops), its performance did not significantly surpass that of VMamba. Particularly in terms of Acc-Top1 and Acc-Top5 accuracy, ViT lagged behind VMamba, possibly due to its reliance on large datasets to maintain performance in large-scale tasks. The VMamba model, with its efficient selective scanning mechanism (S6), achieved higher accuracy and precision while maintaining lower computational complexity (4.85 Gflops). While the ResNet50 model has fewer parameters (23.59M), its performance was inferior to VMamba, especially in Precision and Recall. The DeiT model showed good results in certain metrics, particularly in terms of faster training times per epoch, but its overall predictive performance was still behind that of VMamba and Swin Transformer.

Conclusion of Experiment: The VMamba model demonstrated outstanding performance in large-scale plant disease recognition tasks, surpassing other models in terms of accuracy and computational efficiency. This indicates that the VMamba model holds great potential for applications in agricultural disease identification, especially in scenarios with limited computational resources.

#### 4.3.2 Results and analysis of experiment 2

The results of Experiment 2 are shown in **Table 4**. This experiment primarily evaluates the performance of VMamba on small-sample datasets to verify its generalization capabilities in low-data environments. A key challenge in agricultural disease detection is the difficulty of collecting large-scale, high-quality annotated data in real-world applications. Therefore, we used the In-field small dataset as the experimental object. This dataset contains 3,250 images, divided into three categories: HLB-infected leaves (758 images), magnesium-deficient leaves (739 images), and healthy leaves (1,151 images).This small-sample dataset reflects the practical challenges of data acquisition in real agricultural environments. The images vary in lighting conditions, background complexity, and leaf angles, simulating the complexities of disease detection tasks in small-sample scenarios.

**Table 4:** Results of Experiment 2.

Model	VMamba	ResNet50	DeiT	ViT	Swin Transformer
Params	30.7M	25.56M	5.71M	85.80M	27.53M
Gflops	4.85	4.13	1.08	68.72	4.37
Acc-Top1 (%)	93.21	91.84	82.26	71.59	93.37
Precision (%)	91.74	91.12	82.57	71.68	92.81
Recall (%)	90.49	89.86	80.75	70.16	92.70
F1 Score (%)	90.94	90.26	81.41	70.74	92.69
Time per epoch	59s	241s	34s	94s	228s

The findings indicate that VMamba performed exceptionally well in the small-sample plant disease identification task, surpassing models such as ResNet50, ViT, and DeiT in key performance metrics like Acc-Top1, Precision, Recall, and F1 Score. Additionally, VMamba demonstrated superior efficiency in training time. VMamba achieved an Acc-Top1 accuracy of 93.21%, while the Swin Transformer model slightly outperformed VMamba with 93.37%. Although Swin Transformer had a slight advantage in Acc-Top1, its training time per epoch (228 seconds) was 286% longer than that of VMamba (59 seconds), indicating that VMamba has a significant advantage in computational efficiency. To comprehensively evaluate model performance, we conducted statistical significance tests and calculated confidence intervals for metrics such as accuracy, precision, recall, and F1 score.

Compared to ViT, VMamba’s performance was particularly strong. ViT’s performance dropped significantly on the small-sample dataset, achieving an Acc-Top1 of only 71.59%, much lower than its results on the large-scale PlantVillage dataset. This shows that ViT struggles with small-sample data, whereas VMamba’s efficient architecture helps mitigate the performance degradation associated with small-sample datasets. ResNet50 showed results close to VMamba’s but fell short in both precision and computational efficiency. VMamba’s training time per epoch was 59 seconds, while ResNet50 required 241 seconds, indicating that VMamba is more computationally efficient. Swin Transformer performed well on the small-sample dataset, especially in Acc-Top1 and Precision, slightly outperforming VMamba. This could be attributed to its Window-based Multi-Head Self-Attention (W-MSA) mechanism, which helps extract local features effectively on small datasets (Liu et al., 2021). However, Swin Transformer’s training time per epoch was 228 seconds, which was 286% longer than VMamba’s. Overall, VMamba strikes a better balance between efficiency and accuracy.

Conclusion of Experiment 2: VMamba outperformed ResNet50, ViT, and DeiT on small-sample agricultural disease datasets in terms of both accuracy and computational efficiency. It also performed comparably or better than Swin Transformer on metrics like Acc-Top1 and Time per epoch. VMamba’s efficient selective scanning mechanism and state-space model architecture provide strong adaptability and robustness, maintaining high classification performance while keeping computational complexity low.

#### 4.3.3 Results and analysis of experiment 3

The results of Experiment 3 are shown in **Table 5**. Transfer learning based on the PlantVillage dataset significantly improved VMamba’s performance in the small-sample plant disease identification task. In terms of Acc-Top1, Precision, Recall, and F1 Score, the PV-In-field-VMamba model outperformed PV-In-field-ViT, PV-In-field-ResNet50, and PV-In-field-Swin Transformer. Specifically, VMamba achieved an Acc-Top1 accuracy of 99.81%, significantly higher than ViT’s 93.06% and ResNet50’s 98.26%. In addition to the accuracy advantage, VMamba was also much more efficient. Due to its large parameter size (85.68M) and high computational complexity (16.86 Gflops), ViT took 275 seconds per epoch, while VMamba required only 47 seconds per epoch, with a lower complexity of 4.86 Gflops. Moreover, VMamba showed more stable overall performance across Precision, Recall, and F1 Score after transfer learning. While Swin Transformer performed closely to VMamba in some metrics, it lagged behind in terms of accuracy, especially when handling small-sample disease identification tasks where VMamba’s advantages were more pronounced.

**Table 5:** Results of Experiment 3.

Model	PV-In-field-VMamba	PV-In-field-ViT	PV-In-field-ResNet50	PV-In-field-Swin Transformer
Params	30.7M	85.68M	23.59M	27.53M
Gflops	4.86	16.86	4.13	4.37
Acc-Top1 (%)	99.81	93.06	98.26	96.32
Precision (%)	99.78	91.76	97.79	95.70
Recall (%)	99.78	92.69	98.00	96.17
F1 Score (%)	99.78	92.09	97.89	95.91
Time per epoch	47s	275s	254s	235s

**Figure 9:**
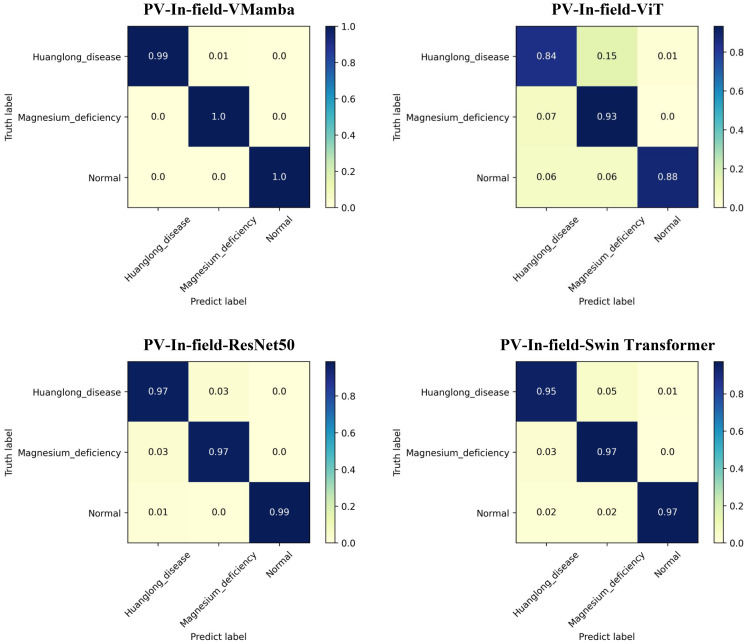
Confusion matrix: Experiment 3.

The Confusion Matrix for each model is shown in **Figure 9**, where PV-In-field-VMamba performed best across all categories. In particular, VMamba showed the highest accuracy in identifying Huanglong disease, while ViT and ResNet50 struggled with misclassification and confusion in recognizing Huanglong disease and Magnesium deficiency. VMamba effectively avoided these errors, indicating stronger distinction and robustness in handling complex agricultural disease categories.

Conclusion of Experiment 3: The transfer learning performance of the VMamba model on small-sample agricultural disease datasets was superior to ViT, ResNet50, and Swin Transformer. VMamba not only excelled in accuracy but also exhibited exceptional computational efficiency, particularly in small-sample disease identification tasks. Through transfer learning, VMamba was able to retain useful features from the pre-trained model while adapting to different datasets with outstanding results.

#### 4.3.4 Results and analysis of experiment 4

The results of Experiment 4 are shown in **Table 6**. The use of the DDPM data augmentation method significantly expanded the small-sample dataset and improved the performance of all models. Specifically, the DDPM-In-field-VMamba model achieved the best results in terms of Acc-Top1, Precision, Recall, and F1 Score, with an Acc-Top1 of 97.93%, far surpassing other models, particularly Swin Transformer (92.83%). In terms of precision and recall, VMamba remained the top performer even after DDPM data augmentation. Although ViT and ResNet50 showed some improvement, they were still unable to outperform VMamba, indicating that VMamba’s architecture continues to excel in handling augmented datasets with outstanding generalization and stability. Additionally, the experiments revealed that DDPM-augmented datasets not only improved model accuracy but also shortened training times per epoch. VMamba took only 49 seconds per epoch, compared to ViT’s 276 seconds and ResNet50’s 253 seconds, achieving a good balance between performance and training efficiency.

**Table 6:** Results of Experiment 4.

Model	DDPM-In-field-VMamba	DDPM-In-field-ViT	DDPM-In-field-ResNet50	DDPM-In-field-Swin Transformer
Params	30.7M	85.68M	23.59M	27.53M
Gflops	4.86	16.86	4.13	4.37
Acc-Top1 (%)	97.93	96.00	96.53	92.83
Precision (%)	97.80	95.14	95.73	92.02
Recall (%)	97.65	95.32	95.92	92.16
F1 Score (%)	97.70	95.23	95.82	92.08
Time per epoch	49s	276s	253s	250s

**Figure 10:**
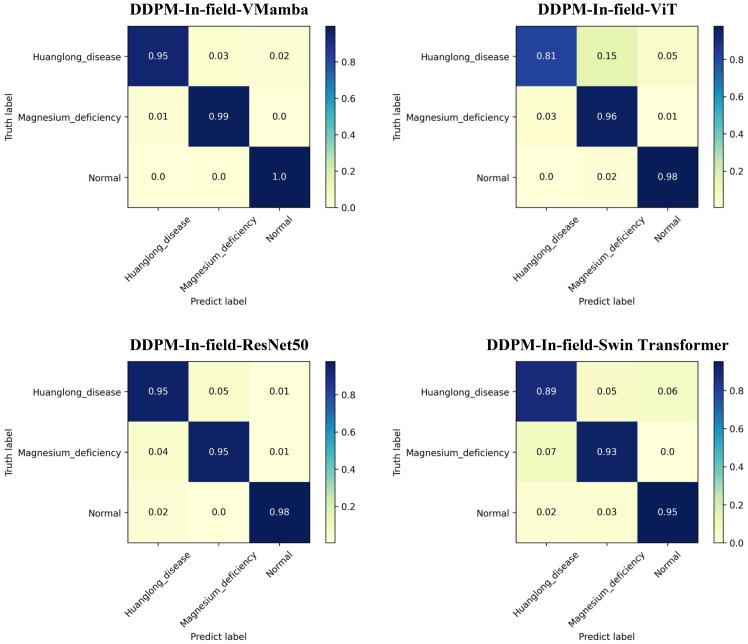
Confusion matrix: Experiment 4.

The Confusion Matrix for each model is shown in **Figure 10**, where DDPM-In-field-VMamba outperformed other models in all categories, particularly in Huanglong disease and Magnesium deficiency recognition. ViT and ResNet50 were more prone to misclassification in these categories, showing less stability when handling complex categories compared to VMamba. The data results were subjected to statistical significance tests, indicating that the Acc-Top1 metric of VMamba was significantly higher than that of other models (a p-value less than 0.05 indicates a significant difference).

Conclusion of Experiment 4: The DDPM data augmentation method significantly improved the model’s performance when working with small-sample agricultural disease datasets. Particularly in the VMamba model, data augmentation after transfer learning led to the best results across metrics such as Acc-Top1, Precision, Recall, and F1 Score, while also greatly improving training efficiency. Compared to other models, VMamba not only achieved higher accuracy but also significantly reduced training time. The comparison of confusion matrices further shows that VMamba exhibited superior accuracy and robustness across different disease categories.

#### 4.3.5 Results and analysis of experiment 5

**Figure 11:**
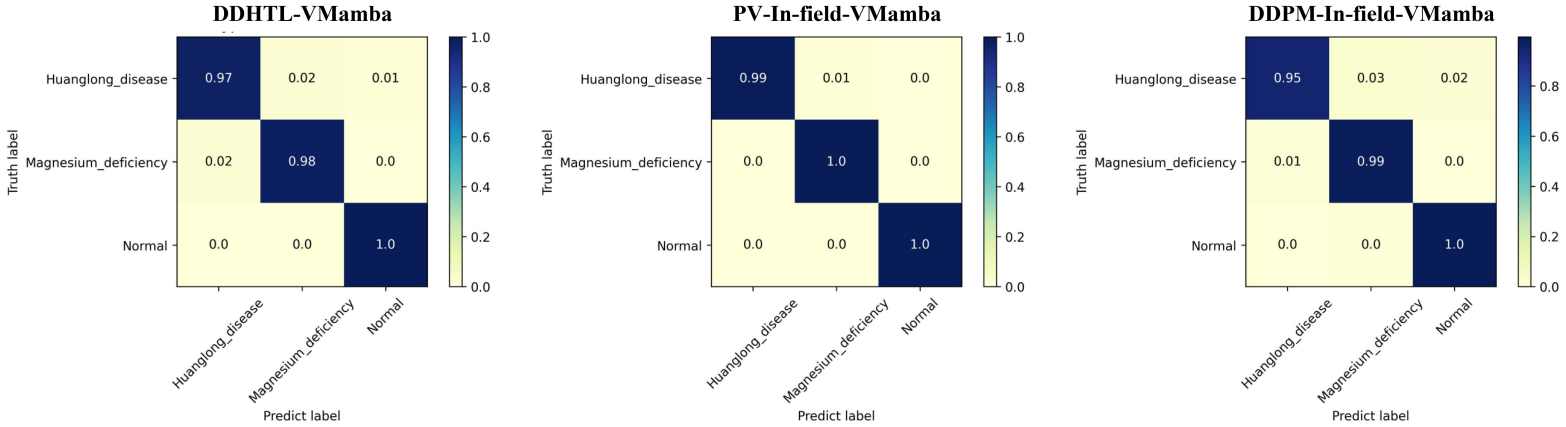
Confusion matrix of Experiment 5.

The results of Experiment 5 are presented in **Table 7**. When using the DDHTL-VMamba training method, the model’s accuracy showed a slight decrease compared to the PV pre-trained transfer learning method, but the time required for the pre-training phase was significantly reduced by 43.88%, which greatly lowered the training cost. Compared to the transfer learning method using DDPM-augmented datasets, the DDHTL-VMamba method showed improvements in model accuracy. The Confusion Matrix is shown in **Figure 11**, where we can observe a clear improvement in identifying Huanglong disease compared to the DDPM-augmented pre-training method. The results demonstrate that the DDHTL-VMamba method can more effectively balance model accuracy and training cost.

**Table 7:** Results of Experiment 5.

Model	DDHTL-VMamba	PV-In-field-VMamba	DDPM-In-field-VMamba	Common data enhancement-VMamba
Params	30.7M	30.7M	30.7M	30.7M
Gflops	4.86	4.86	4.86	4.86
Acc-Top1 (%)	99.434	99.811	97.925	92.830
Precision (%)	99.338	99.776	97.797	92.554
Recall (%)	99.342	99.781	97.650	91.819
F1 Score (%)	99.333	99.778	97.704	92.109
Pretrained time	14,496s	25,830s	12,200s	—

## 5 Discussion

Through a series of experiments using the VMamba model on different datasets and training methods, this study found that VMamba exhibits outstanding performance in agricultural disease identification, especially on small-sample datasets. Compared to other popular vision models such as ResNet50, ViT, and Swin Transformer, VMamba consistently outperformed them on several key metrics (Acc-Top1, Acc-Top5, Precision, Recall, F1 Score). This was particularly evident in small-sample conditions and when transfer learning and DDPM data augmentation were applied, where VMamba’s performance further stood out.

In Experiment 1, the VMamba model demonstrated superior performance on the PlantVillage dataset. Compared to models like ResNet50, ViT and Swin Transformer, VMamba’s architecture, based on state-space modeling, was better at handling complex visual features. In Experiment 2, VMamba continued to outperform ResNet50, DeiT, and ViT on the In-field small dataset, showcasing its effectiveness in small-sample data scenarios. This advantage can be attributed to VMamba’s selective scanning mechanism (S6), which maintains a global receptive field while reducing computational complexity, thereby enhancing the model’s generalization ability. Although ResNet50 performs well on large-scale datasets, it struggles with generalization on small-sample datasets. Similarly, the ViT model, due to its high parameter count and reliance on large datasets, performed poorly when data was limited. In Experiment 3, the introduction of transfer learning significantly improved VMamba’s performance on small-sample datasets. After pre-training on the PlantVillage dataset, the model could learn general features that maintained high recognition accuracy when transferred to small-sample datasets. This finding aligns with existing literature, which indicates that transfer learning can greatly enhance model performance on small datasets (Zhao, 2017; Zhao et al., 2020). In Experiment 4, the DDPM diffusion model was used to generate additional images, further improving the model’s recognition capability. The augmented dataset provided richer features for the small-sample data, increasing the model’s robustness. The results showed that VMamba performed the best across multiple metrics after DDPM augmentation, confirming the effectiveness of data augmentation for small-sample challenges. In Experiment 5, the DDHTL-VMamba training method was proposed, which pre-trains on a combination of DDPM-augmented and original datasets and then transfers to the In-field small dataset. The results indicate that this method effectively balances model accuracy with reduced pre-training time, significantly lowering training costs. Compared to traditional data augmentation methods (which include transformations such as rotation, scaling, cropping, flipping, and noise addition like Gaussian and salt-and-pepper noise), both DDPM and DDHTL demonstrated superior performance. This shows that the DDHTL-VMamba training method is ideal for agricultural disease identification tasks where balancing training cost and model performance is critical.

**Figure 12:**
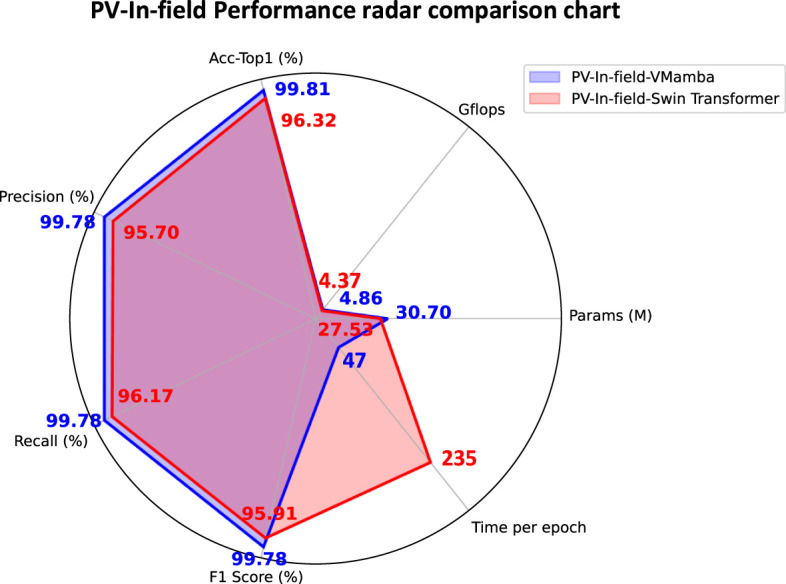
PV-In-field performance radar comparison chart.

The VMamba model addresses the limitations of traditional The VMamba model addresses the limitations of traditional ViT and ResNet models in handling small-sample data and complex agricultural background data, demonstrating superior performance. Compared to Swin Transformer, proposed by Liu et al. (2021), which is currently the state-of-the-art (SOTA) method, the VMamba model shows better performance on complex agricultural images and small-sample agricultural data, as shown in **Figure 12**. Swin Transformer is a hierarchical architecture based on Transformers, which significantly reduces computational complexity by introducing local window multi-head self-attention mechanisms (W-MSA) while retaining feature extraction capabilities. Its W-MSA mechanism divides images into fixed-size, non-overlapping windows, making it difficult to capture global dependencies across windows, thus limiting its performance in agricultural disease identification tasks. For example, in citrus leaves infected with Huanglongbing (HLB), lesions may be scattered across the leaf tip, leaf margin, and petiole regions. Window partitioning can disrupt the association between these areas, reducing the model’s overall perception of the disease.

The VMamba model, based on state-space models (SSM), uses a selective scanning mechanism (S6) to achieve global context modeling through linear scanning. VMamba processes image sequences independently from four directions and integrates multi-directional context information during the merging stage. This design allows it to capture various distribution patterns of leaf diseases, whereas the single-direction window partitioning of Swin Transformer struggles to adapt to such complex morphologies. Experiment 3 shows that VMamba’s Acc-Top1 is 3.49% higher than Swin Transformer (99.81% vs. 96.32%) in complex background interference, validating its advantage in global modeling. In small-sample datasets, the W-MSA mechanism of Swin Transformer initially provides a slight accuracy advantage over VMamba in early training stages. However, after DDPM data augmentation and pre-training optimization, VMamba’s global attention-aware capability surpasses that of Swin Transformer, ResNet50, and ViT models.

Although the VMamba model showed excellent performance in this study, there are some limitations. First, while data augmentation and transfer learning significantly improve small-sample dataset performance, these methods rely on high-quality augmented data and pre-trained models. If the original data quality is poor, it may negatively affect the model’s performance. Moreover, this study only experimented with disease identification in citrus plants; future research should further evaluate VMamba’s applicability in other crops and disease identification tasks.

## 6 Conclusions and future work

### 6.1 Limitations

VMamba performs exceptionally well in high-performance computing environments, but its deployment on mobile and edge computing devices presents certain challenges. These devices typically have limited computational power and memory, which may lead to issues such as memory overflow and high computational latency when running VMamba directly. Although VMamba reduces computational complexity through its S6 mechanism, with a computational cost of 4.85 Gflops, which is significantly lower than that of mainstream models like ViT (16.86 Gflops), its real-time performance on edge devices—such as frame rate and power consumption—still requires further testing. In practical applications like farmland monitoring, additional optimizations may be necessary, such as model pruning, to reduce the model size and computational load.

The current experiments were primarily validated on citrus diseases. While VMamba demonstrated excellent transfer learning performance on multi-crop datasets (e.g., PlantVillage) and small-sample datasets (e.g., In-field small dataset), its transferability to other crops such as rice and wheat remains to be further explored. The small-sample dataset used in this study was sourced from real agricultural environments and exhibits certain regional characteristics. Although we adhered to ethical standards during data collection to ensure the legality and accuracy of the data, and took measures to minimize bias, future research should aim to collect image data from different regions and crop types. This will help validate VMamba’s performance across diverse conditions and reduce errors.

### 6.2 Conclutions

In this study, we introduced the emerging VMamba vision backbone model for the task of agricultural plant disease identification, including small-sample plant disease recognition. Experimental results demonstrated that, whether applied to large datasets like PlantVillage or optimized small-sample agricultural disease datasets, VMamba consistently outperformed popular models such as ResNet50, Vision Transformer (ViT), and Swin Transformer in terms of accuracy for plant disease identification. These results verify VMamba’s excellent performance in the field, offering a novel approach for agricultural plant disease recognition. Additionally, we proposed the DDHTL-VMamba training method for small-sample agricultural disease datasets. Compared to transfer learning using large datasets, this method largely maintained the accuracy of plant disease identification while significantly reducing the time required for pre-training, thereby providing a more efficient and balanced solution.

### 6.3 Future work

Future research could focus on evaluating VMamba’s performance on larger and more diverse field datasets, particularly in real-world agricultural environments where crop conditions and disease manifestations are often more complex. Further improvements could be made by integrating other data augmentation techniques or optimizing transfer learning strategies to enhance the model’s generalization capabilities on small-sample datasets. Another promising direction would be exploring the application of VMamba in multimodal data (e.g., spectral images and sensor data) to broaden its potential in precision agriculture.

## Data Availability

The original contributions presented in the study are included in the article/supplementary material. Further inquiries can be directed to the corresponding author.
